# Neonatal ibuprofen exposure and bronchopulmonary dysplasia in extremely premature infants

**DOI:** 10.1038/s41372-019-0444-4

**Published:** 2019-08-07

**Authors:** Xueyu Chen, Xiaomei Qiu, Panpan Sun, Yanqing Lin, Zhifeng Huang, Chuanzhong Yang, Frans J. Walther

**Affiliations:** 1 Department of Neonatology, Affiliated Shenzhen Maternity & Child Healthcare Hospital, Southern Medical University, Shenzhen, China; 2 Department of Pediatrics, David Geffen School of Medicine, University of California Los Angeles, Los Angeles, CA USA; 3 Los Angeles Biomedical Research Institute at Harbor‐UCLA Medical Center, Torrance, CA USA

**Keywords:** Risk factors, Paediatrics

## Abstract

**Objective:**

To evaluate the association of ibuprofen exposure with the risk of bronchopulmonary dysplasia (BPD) in extremely premature infants.

**Study design:**

This was a retrospective study of all extremely premature infants admitted to a tertiary unit from 2016 to 2018.

**Results:**

A total of 203 extremely premature infants were included in this study. The rate of BPD was significantly higher in infants with early exposure to ibuprofen (42.5%) compared to infants with no exposure (21.6%, *P* = 0.001). After adjusting for covariates, the risk of BPD was associated independently with ibuprofen exposure (odds ratios (OR) 2.296, 95% confidence interval (CI): 1.166–4.522, *p* = 0.016). Further analysis showed a trend towards higher risk of BPD in infants with successful patent ductus arteriosus (PDA) closure after ibuprofen treatment (32.3%) compared to non-treated infants (20.2%, *p* = 0.162).

**Conclusion:**

Our findings suggest that ibuprofen exposure may contribute to the occurrence of BPD in extremely preterm infants.

## Introduction

Extremely premature infants (gestational age ≤ 28 weeks) are at high risk of bronchopulmonary dysplasia (BPD) [1]. The prevalence of BPD in extremely premature infants remains stable at around 40% [2, 3] despite great advances in prenatal care in recent years. BPD is characterized by arrested alveolar and vascular development [4]. As a result, survivors of BPD suffer from short and long-term complications from neonatal infection to adolescent asthma and chronic obstructive pulmonary disease (COPD) in adult life [5].

Many efforts have been devoted to identifying factors that deteriorate lung development after extremely preterm birth [6]. Ibuprofen is one of the nonsteroidal anti-inflammatory drugs (NSAIDs) and is extensively used for the treatment of patent ductus arteriosus (PDA) [7, 8]. Recently, the adversity of early exposure to ibuprofen in newborns has drawn much attention [8–10], among which pulmonary arterial hypertension (PAH) is a significant problem [11–13]. Notably, in vitro and in vivo experiments corroborate that ibuprofen inhibits angiogenesis [14, 15]. Blunted angiogenesis is exactly the underlying mechanism of both neonatal PAH and BPD [16].

The association between early exposure to ibuprofen and BPD remains controversial. Several studies reported that ibuprofen was not associated with the development BPD (as exploratory outcome) [17, 18]. Ohlsson et al. found no difference in the prevalence of BPD in infants with or without ibuprofen treatment [18]. Mitra et al. found a reduced risk of BPD after ibuprofen treatment compared to placebo [17]. However, a large proportion of the controls in those studies actually received treatment for PDA [19], leading to concerns about the accuracy of the conclusion in those studies. Therefore, this study aims to investigate the association of early ibuprofen exposure with BPD in a cohort of extremely premature infants. This study also compares the prevalence of BPD in subgroups stratified by PDA status.

## Methods

This retrospective study was conducted at the neonatal intensive care unit (NICU) of Shenzhen Maternal & Child Healthcare Hospital between January 2016 and July 2018. All neonates born before 28 weeks’ gestation at our center were eligible for this study. During the study period, oral ibuprofen (Melian, Shanghai Johnson & Johnson Pharmaceuticals Co., Ltd, Shanghai, China) was used only for hemodynamic significant PDA. A course of treatment was defined as an initial dose of 10 mg/kg body weight on the first day followed by 5 mg/kg on the second and third day. This study was approved by the Shenzhen Maternity & Child Healthcare Hospital Institutional Ethical Committee and the requirement for informed consent was waived by the committee.

Extreme prematurity was defined as delivery at a gestational age ≤ 28 weeks. An echocardiogram was routinely performed within 72 h after birth. A hemodynamically significant PDA (hsPDA) was diagnosed by the presence of a ductus with a diameter > 1.5 mm, left atrial inner diameter/aortic root (LA/AO) ≥ 1.4, combined left to right shunt [20]. Infants with a hemodynamically significant PDA received at least one course of oral ibuprofen in absence of contraindications such as a low platelet count (<50,000 per mL), oliguria, bloody vomit, necrotizing enterocolitis (NEC) and renal deficiency. Another echocardiogram was performed within 48 h after each treatment course. BPD was diagnosed when supplemental oxygen was required at 36 weeks postmenstrual age (PMA) or discharge [21].

Data were retrieved from the electronic medical record, including maternal complications such as gestational hypertension and gestational diabetes mellitus (GDM), maternal infection, antenatal steroid treatment, delivery methods, gestational age (GA), birth weight (BW), Apgar score, gender, surfactant treatment, ventilation mode, PDA and information about ibuprofen treatment.

## Statistics

The sample size calculation was based on the prevalence of BPD in our unit. The rate of BPD in ibuprofen treated infants and not treated ones were around 40% vs 20%. At 80% power and α = 0.05, 80 infants in each group would be sufficient to detect a significant difference (PASS, Version 11, NCSS, LLC, Utah). The Shapiro–Wilk test was used to evaluate the normality of continuous variables and the independent *t*-test (two sides) and the Mann–Whitney U test to analyze continuous variables according to the normality. Chi-square or Fisher’s exact tests were used to compare categorical data, as appropriate. Potential risk factors for BPD were first assessed in a univariable regression model (binary regression). Those factors significantly associated with BPD were then inserted into a multivariable logistic regression model (Forward) to evaluate their independence in relation to BPD. Odds ratios (OR) and 95% confidence interval (CI) were calculated using univariable and multivariate regression analysis. The analysis was performed using SPSS statistical software version 24.0 (IBM Corporation, NY).

## Results

**Fig. 1:**
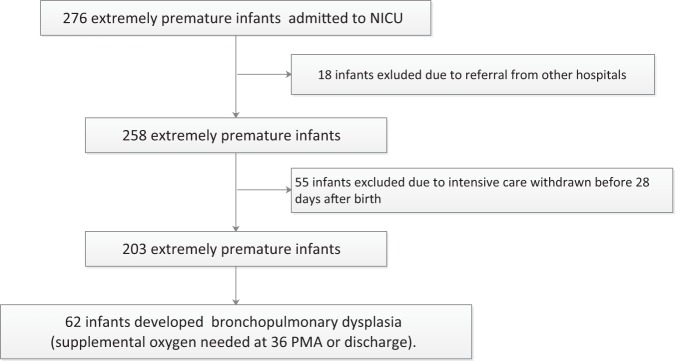
Flowchart of case selection and analyses

A total of 276 extremely premature infants were admitted to our NICU during the study period. In total 18 infants were excluded because of referral from other hospitals and incomplete data. A total of 55 infants were also excluded due to withdrawal from intensive care before 28 days after birth by parents’ choice. The remaining 203 extremely premature infants were included in this study (Fig. 1). BPD was diagnosed in 62 (30.5%) infants. The clinical characteristics by ibuprofen exposure are summarized in Table 1.

**Table 1:** Clinical characteristics by ibuprofen exposure

Variable	no ibuprofen (*n* = 116)	ibuprofen (*n* = 87)	P value
GDM	13 (11.2%)	10 (11.5%)	0.949
Gestational hypertension	3 (2.6%)	4 (4.6%)	0.465
Maternal infection	16 (13.8%)	15 (17.2%)	0.499
Fetal distress	4 (3.4%)	5 (5.7%)	0.502
Antenatal steroid treatment	86 (74.1%)	70 (80.5%)	0.224
Cesarean section delivery	26 (22.4%)	23 (26.4%)	0.507
Gestational age at birth (wk)	27.0 (26.0–27.3)	26.2 (24.6–27.2)	0.012
Birth weight (gr)	943 ± 189	866 ± 204	0.006
Male	68 (58.6%)	54 (62.1%)	0.620
Intubation	48 (41.4%)	53 (60.9%)	0.006
Apgar score at 1 min	7 (5–9)	5.5 (5–8)	0.035
Apgar score at 5 min	10 (9–10)	10 (9–10)	0.165
Surfactant treatment	89 (76.7%)	82 (94.2%)	0.001
BPD	25 (21.6%)	37 (42.5%)	0.001
IVH	33 (28.4%)	31 (35.6%)	0.276
Gastrointestinal perforation	11 (9.5%)	13 (14.9%)	0.230
Intervened ROP	16 (13.8%)	25 (28.7%)	0.008

Data were displayed as mean ± SD, median (interquartile range) or number (percentage). *GDM* gestational diabetes mellitus, *BPD* bronchopulmonary dysplasia (oxygen needed at PMA 36 weeks), *IVH* intraventricular Hemorrhage, *ROP* retinopathy of prematurity, intervened ROP was defined as ROP requiring either laser therapy or medication injection

The univariable analysis showed that the occurrence of BPD was associated with gestational age, birth weight, intubation, 1- and 5-min Apgar score, surfactant treatment, hsPDA and ibuprofen exposure. These risk factors were subsequently entered into the multivariable regression model (Table 2). We found that the occurrence of BPD was independently associated with gestational age (OR 0.498, 95% CI: 0.374–0.662, *P* < 0.000) and ibuprofen exposure (OR 2.296, 95% CI: 1.166–4.522, *p* = 0.016).

**Table 2:** Univariate and multivariate logistic regression analysis of selected variables associated with BPD

Variables	Univariate		Multivariate	
	RR (95% C.I.)	*P* value	RR (95% C.I.)	*P* value
Ibuprofen exposure	2.694 (1.458, 4.975)	0.001	2.296 (1.166, 4.522)	0.016
Gestational age	0.480 (0.364, 0.633)	< 0.001	0.498 (0.374, 0.662)	< 0.001
1 min Apgar Score	0.791 (0.684, 0.915)	0.002	−	0.118
5 min Apgar Score	0.747 (0.565, 0.986)	0.009	−	0.971
Birth weight	0.996 (0.994, 0.998)	< 0.001	−	0.236
Intubation	2.385 (1.286, 4.423)	0.005	−	0.686
Surfactant	4.500 (1.309, 15.464)	0.010	−	0.356
HsPDA	2.929 (1.574, 5.452)	0.001	−	0.857

BPD was diagnosed when supplemental oxygen required at 36 weeks PMA. HsPDA (hemodynamically significant PDA) was defined as the presence of a ductus with a diameter > 1.5 mm, LA/AO (left atrial inner diameter/aortic root) ≥ 1.4, combined left to right shunt

In our cohort, 42.8% (87/203) infants were given at least one course of ibuprofen for the treatment of PDA, yielding a successful closure rate of 35.6% (31/87) within 48 h after completion of the treatment course. The median age for the first dose of ibuprofen was 6.5 (4, 9.25) day, with median feeding volume 1 (0.5, 2.5)ml per feeding. 23.0% (20/87) infants receiving ibuprofen treatment suffered from feeding intolerance or gastric complications within 72 h after the first dose.

**Fig. 2:**
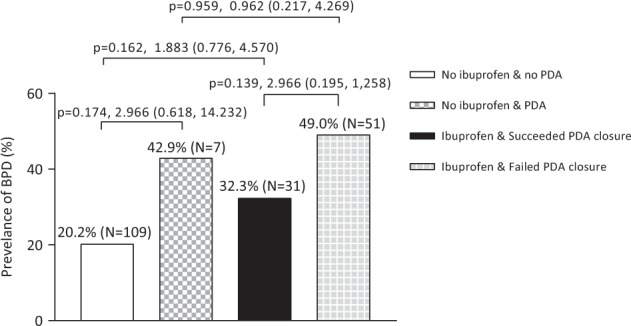
Analysis of ibuprofen exposure and the risk of BPD in sub-groups with variate ductal status. Comparisons were performed between groups with a bridge connecting them. Prevalence of BPD, sample size, *p*-value, OR and 95% C.I. was indicated on the bars and bridges

To eliminate the confounding of PDA, we further analyzed the association of ibuprofen with the risk of BPD by stratifying ductal status. We found that among infants with a closed ductus (either by itself or by ibuprofen treatment), the frequency of BPD in infants with early ibuprofen exposure (32.3%) was not significantly higher than infants receiving no ibuprofen (20.2%; OR 1.883, 95%CI 0.776–4.570, *p* = 0.162; Fig. 2). A similar finding of the prevalence of BPD was found in infants with an open ductus (either treated or not treated with ibuprofen), 49.0% and 42.9%, respectively, (OR 0.962, 95% CI 0.217–4.269, *p* = 0.959; Fig. 2). Furthermore, PDA increased (not significantly) the risk of BPD in infants not receiving ibuprofen (42.9% and 20.2%) and receiving ibuprofen (49.0% and 32.3%, Fig. 2).

The clinical characteristics of infants during ibuprofen treatment were described in Table 3. The median age, feeding volume and rate of a single course of treatment were similar between the infants developing BPD and those not (7th vs 6th day, *p* = 0.670; 2 vs 1 ml/feeding, *p* = 0.995; 62.2% vs 70%, *p* = 0.265, respectively). Infants who developed BPD, compared to infants who did not, show no significant differences in the rate of feeding intolerance (18.9% vs 26%, *p* = 0.439) or the rate for PDA surgical ligation (18.9% vs 8%, *p* = 0.140).

**Table 3:** Variates about ibuprofen treatment and their association with BPD

	BPD (*n* = 37)	Non-BPD (*n* = 50)	OR (95%CI)	*P* value
Age of the first dose of ibuprofen (Day)	7 (3.5, 10.5)	6 (4, 9)	0.982 (0.901, 1.069)	0.670
Feeding volume at the first dose (ml/feeding)	2 (1, 3)	1 (0.5, 2.375)	0.995 (0.907, 1.091)	0.995
Feeding intolerance	7 (18.9%)	13 (26%)	0.664 (0.235, 1.874)	0.439
Single course of ibuprofen	23 (62.2%)	35 (70%)	1.528 (0.725, 3.219)	0.265
Multiple course of ibuprofen	14 (37.8%)	15 (30%)	−	−
PDA surgical ligation	7 (18.9%)	4 (8%)	2.683 (0.723, 9.962)	0.140

Feeding intolerance, including gastric residuals, abdominal distension, vomiting and gastric bleeding occurred within 72 h after the first dose of ibuprofen

## Discussion

This study showed that BPD is approximately 2-fold more likely to occur in infants exposed to ibuprofen during the first days of life. This suggests that early ibuprofen exposure in neonatal may contribute to the occurrence of BPD in extremely premature infants.

The association of ibuprofen with the development of BPD has been a great interest of neonatologists. In two systematic and meta-analysis, Ohlsson et al. and Mitra et al. reported no effect of ibuprofen on BPD development [17, 18], which is inconsistent with the findings in this study and may be due to differences in the control group (infants with open ductus in their studies), study population (GA < 37 weeks in their studies) and the diagnostic standard of BPD. In addition, up to 85% (an average of 50%) of the control infants in previous RCTs actually received treatments of PDA in the later study period. Therefore the conclusion of the systematic and meta-analysis can only be made between the early- and late-treated PDA instead of treated and non-treated PDA [19]. Fortunately, the awareness of this limitation in previous RCTs is well recognized and new trials on PDA management have been initiated, such as Baby-Oscar (https://www.npeu.ox.ac.uk/baby-oscar), PDA trial (https://clinicaltrials.gov/ct2/show/NCT03456336) and Benedictus (https://Neonatologynetwork.eu/studies/beneductus). The completion of these trials would provide solid evidence of an association between ibuprofen exposure and BPD.

It’s unclear why early exposure to ibuprofen is associated with an increased risk of BPD. BPD is characterized as arrested alveolar and vascular development [22]. *Ex vivo* experiments substantiate that ibuprofen inhibits angiogenesis by disturbing the tube formation ability of human umbilical vein endothelial cell (HUVEC) [14]. In newborn rats, ibuprofen significantly diminishes neovascularization [15]. Clinical studies demonstrate a higher frequency of PAH after ibuprofen treatment in very low birth weight infants (<1500 g) [23]. Collectively, the anti-angiogenetic effect of ibuprofen might be the underlying mechanism of its effect on BPD, especially in extremely premature infants who are more vulnerable to blocked angiogenesis. However, multiple courses of ibuprofen exposure did not significantly increase the risk of BPD (this study), which may be owing to the small sample size.

However, further analysis of the association between ibuprofen and BPD among subgroups did not confirm the findings in the whole cohort. In infants with a closed ductus, the prevalence of BPD in infants without ibuprofen treatment (*N* = 109, 20.2%) is not significantly lower than in infants exposed to ibuprofen (*N* = 31, 32.3%). A similar result was found in infants with an open ductus (42.9% vs 49.0%). The discrepancy of these findings may be attributed to the relatively small sample size of the subgroups. Notably, the close rate of ibuprofen on PDA in the current study (35.6%) is lower compared with other studies, where the closure is about 56.7%–78.8% [17, 24, 25]. This difference in closure rate of PDA by ibuprofen may be explained by a different route of administrating ibuprofen, study population and various starting age in those studies. Both younger gestational age and later treatment contributed to the failure of pharmaceutical closure of PDA. In the study from Bagnoli et al., 50% of infants with birth weight ≤ 750 g required surgical ligation [24].

The main limitation of our study, besides the retrospective nature, is the relatively limited sample size, especially in the subgroups. Besides, infants with non-treated PDA are usually the ones with abdominal complications, which may be associated with the occurrence of respiratory disorders in infants themselves. Therefore, our conclusion should be interpreted with care when extrapolating to the general population.

In summary, early exposure of ibuprofen may be associated with an increased risk of BPD in extremely premature infants. Larger cohorts are required to investigate the possible negative effects of ibuprofen exposure on the developing premature lung.
